# Sex Differences in Severity and Recovery Following Mild Traumatic Brain Injury: A Systematic Review

**DOI:** 10.3390/brainsci16010077

**Published:** 2026-01-06

**Authors:** Shanika Arachchi, Ed Daly, Anushree Dwivedi, Lisa Ryan

**Affiliations:** 1 Department of Sport, Exercise and Nutrition, Atlantic Technological University, H91 T8NW Galway, Ireland; shanika.arachchi@research.atu.ie (S.A.); ed.daly@atu.ie (E.D.); 2 Department of Mechanical and Industrial Engineering, Atlantic Technological University, H91 T8NW Galway, Ireland; anushree.dwivedi@atu.ie

**Keywords:** mild traumatic brain injury (mTBI), sports-related concussion (SRC), sex differences, cognitive impairment, symptom severity, recovery, neuroimaging

## Abstract

**Background**: Sex-based variations in brain structure, hormonal balance, and neurochemistry may influence symptom presentation and recovery after mild traumatic brain injury (mTBI). This systematic review investigated sex-related differences in mTBI severity, symptoms, and recovery outcomes across different injury mechanisms. **Methods**: This review followed PRISMA 2020 guidelines and was registered with PROSPERO (CRD420251011379). Searches were conducted in PubMed, SPORTDiscus, Web of Science, and Scopus for articles published between 2000 and 2024. Eligible studies included adults (≥18 years) diagnosed with mTBI or concussion (Glasgow Coma Scale 13–15) with quantifiable outcome data for both sexes. Data extraction and quality assessment followed the JBI critical appraisal tools. **Results**: Forty-one studies involving 12,382 participants (7021 males; 5361 females) met the inclusion criteria. Female participants reported a greater symptom burden, higher pain intensity, and longer recovery times for gait abnormalities and return to activity compared with males. Neuroimaging studies showed more extensive white matter alterations in females, whereas males displayed greater reductions in cerebral blood flow. Cognitive and neurosensory outcomes revealed poorer cognitive performance, slower reaction times, and higher rates of vestibular–ocular and visual abnormalities in females. A limited number of studies explored electrophysiological measures, indicating sex-based differences in early brain responses to emotional stimuli. **Conclusions**: Sex plays an important role in symptom presentation and recovery after mTBI. Female patients demonstrate heightened vulnerability across several clinical domains, likely due to biological and neurochemical differences. Recognising these sex-specific patterns can support more targeted diagnostic and rehabilitation strategies. Future research should further explore the structural and biochemical mechanisms underlying these differences to improve precision in mTBI management.

## 1. Introduction

Approximately 42 million people worldwide annually experience mild traumatic brain injury (mTBI), including concussion [1]. The occurrence of mTBIs is common among the civilian population [2], sports community [3], and military personnel [4]. Sudden lateral or torsional movements of the brain inside the skull due to external force to the head or body may induce biochemical reactions in the brain tissue. This biochemical process causes a massive release of neurotransmitters (like glutamate) and ionic flux (potassium and sodium/calcium). This process causes a surge in energy demand and a subsequent energy crisis, leading to acute and subacute changes in cellular physiology with impaired blood flow and potential long-term cognitive impact [5]. These alterations cause the cognitive [6], sensory [7], electrophysiology [8], and gait and balance [9] functions of the patient. The untreated or not fully recovered mTBI conditions may increase the risk for neurodegenerative diseases in later life.

Clinical signs immediately following an mTBI may include loss of consciousness, altered mental condition, amnesia, incoordination of motor control, seizures; some post-injury symptoms include acute alterations in mental status, headaches, dizziness, balance or vision problems, light or noise sensitivity, mental fog, difficulty concentrating, fatigue, and emotional distress [10]. Cognitive impairments are one of the commonly observed symptoms following an mTBI, and persistent conditions can negatively affect the quality of life of individuals even after full recovery [11]. The axons are the long, slender projections of neurons, which conduct electrical impulses away from the cell body; these axons are more vulnerable to damage during an mTBI. The axonal damage occurs due to the shearing force induced during the injury, resulting in diffuse axonal injury (DAI). DAI plays a major role in cognitive impairments after an mTBI [12]. These cognitive impairments can be seen as cognitive deficits, emotional distress, or somatic complaints [13]. However, most symptoms differ due to person-specific factors such as age, sex, pre-injury conditions, and pre-existing health conditions [14,15].

Clinical studies demonstrate that sex and age are the primary patient-specific factors influencing symptom identification, diagnosis, and post-injury treatments in patients with mTBI [16]. Moreover, female sex is identified as one of the main potential risk factors in addition to pre-concussion history, Glasgow Coma Scale (GCS) of [13,14,15], aetiology of assault, preinjury psychological history, etc. [17]. In addition, female athletes have potentially greater injury burden [18] and higher post-concussion scores [19] compared to their male counterparts. Biologically, females report higher levels of physiological stress compared to males [20]. These sex-based differences may be observed due to the structural variations in the male and female brain tissue, in addition to the biochemical variation with respect to the differences in hormonal and neurochemical levels. Previous studies on mTBI have been based on male participants, and limited studies have included female participants [21,22]. A significant knowledge gap persists regarding the impact of sex differences on mTBI symptom reporting, diagnosis, and treatment interventions, highlighting the need for further research. Therefore, the aim of this systematic literature review (SLR) was to investigate the current state of the art of the sex-based variations in mTBI severity and recovery. Identifying these variations may help to improve the treatment interventions and post-injury recovery.

## 2. Materials and Methods

### 2.1. Literature Search

The authors conducted an initial database search in November 2024 using the following keywords with the Boolean terms: “(Concussion OR mTBI OR Mild Traumatic Brain Injury OR sports concussion OR Accidents OR Falls OR Whiplash) AND (Sex OR Gender OR male OR Man OR Men OR Female OR Women OR Woman)”. The search terms, synonyms and related terms are shown in Table 1. The search was conducted in four databases (PubMed, SPORTDiscus, Web of Science, and Scopus) for articles published from 2000 to 2024. This systematic review follows the Preferred Reporting Items for Systematic Review and Meta-Analysis guidelines [23]. This systematic review was registered under PROSPERO 2025 CRD420251011379.

### 2.2. Inclusion and Exclusion Criteria

The inclusion and exclusion criteria of this SLR were based on the PICO (Population, Intervention, Comparator, and Outcome) framework, which was agreed upon by all the authors. The studies were included if they satisfied the PICO inclusion criteria as follows. (1) The population included all the healthy male and female participants ages 18 and over who were diagnosed with mTBI/concussion (GCS of 13–15). (2) The intervention was based on the injury mechanism, which included contact/non-contact sports, trauma, collision, head accelerations (whiplash), or blast-related injuries. (3) The inclusion outcome was based on the concussion/mTBI diagnostic and prognostic outcomes with quantifiable data observed and reviewed for the male and female patients. The outcome measures included the mTBI incident and recovery stage related to cognitive, motor control, visual, and vestibular impacts along with the findings from brain imaging. (4) The inclusion comparator was based on the outcome comparison against the healthy participants in addition to sex-based variations. Furthermore, the biologically defined sex/gender as male or female was considered in this review study.

The studies were excluded if they did not satisfy the inclusion criteria for population, intervention, comparator, and outcome. Studies were excluded if the population was individuals under 18 years old, human or animal cadavers, animals, or cell studies. In addition, if the intervention was moderate to severe TBIs with a GCS index below 12 or inconclusive or unspecified brain injuries those studies were excluded. Furthermore, if the injury mechanism was complicated resulting in severe injuries in addition to a brain injury, those studies were excluded due to the complexity of the injury severity. Studies were excluded if the participants had pre-existing health conditions including neurodegenerative diseases or other drug interventions. Furthermore, studies were excluded if the outcome was based on the validation study for a treatment or protocol intervention. All the non-English and narrative study designs were excluded.

### 2.3. Selection Process

All search results retrieved were exported into an EndNote 21.3 (Philadelphia, PA, USA) reference management software library for eligibility screening. The initial screening of all the titles and abstracts was conducted to remove the duplicates. Then, the remaining titles were coded according to the inclusion criteria. The ineligible articles were moved to a different library according to the PICO exclusion investigation. The PICO protocol was developed precisely upon the agreement of all the authors prior to the initial literature search. A random selection of 20 reports was shared among all the co-authors to assess the repeatability and the reproducibility of the selection criteria. All the co-authors agreed with the selection criteria. Next, the first author screened the included full-text articles according to the inclusion and exclusion criteria. The inclusion criteria were further refined with the agreement of the co-authors. We decided to exclude the articles focused on model-based outcomes (artificial intelligence or machine learning-based, etc.), complicated traumas, validation studies (including frameworks, drug or nutrition interventions, instruments, etc.), and case reports (due to no comparison).

After that, the first author continued to full-text screening and coding according to the inclusion criteria. Then, the finally selected articles were shared among the co-authors for further assessment of whether they include or exclude the papers. All the co-authors agreed on the selected articles to include in this SLR.

### 2.4. Data Extraction

The data were extracted by the first author using the Excel work platform. The following information was extracted from the selected articles: title, DOI, author, year of publication, population size, outcome measure, and key findings related to sex-based variations. The data extraction process was verified by co-authors who randomly selected a subset for cross-checking.

### 2.5. Risk of Bias (RoB) Assessment

The selected articles included cohort studies, cross-sectional studies, case–control studies, and systematic reviews. Hence, the quality of the selected articles was assessed using JBI’s critical appraisal tool [24,25]. JBI is an international research organisation based in the Faculty of Health and Medical Sciences at the University of Adelaide, South Australia. JBI tools for cohort studies, cross-sectional studies, case–control studies, and systematic reviews were used to evaluate the quality of the study methodology and the possible bias in the study design, conduct, and analysis. All questions in the JBI’s critical appraisal tools were organized into an Excel spreadsheet for the ease of analysis. For each study design, the appraisal tool checklist was filled out. Reasons for exclusion were clearly tabulated in the same Excel spreadsheet. Each co-author performed a pilot test separately using a copy of the embedded Excel spreadsheet to verify the assessment criteria.

A quality assessment scaling system was defined as follows, “Yes” (1 Point) “No” (0 Points), and “Unclear/NA” (0.5 Points). The total points scored by each study were presented as a percentage for easier comparison. All co-authors reviewed the final Excel spreadsheet and agreed with the reasons for including or excluding each study. If the quality assessment percentage fell below 50%, the article was removed from the selection, resulting in one article being eliminated. In addition, a systematic review article was replaced with two hand-searched articles included in the same paper, which satisfy the inclusion criteria.

## 3. Results

**Figure 1:**
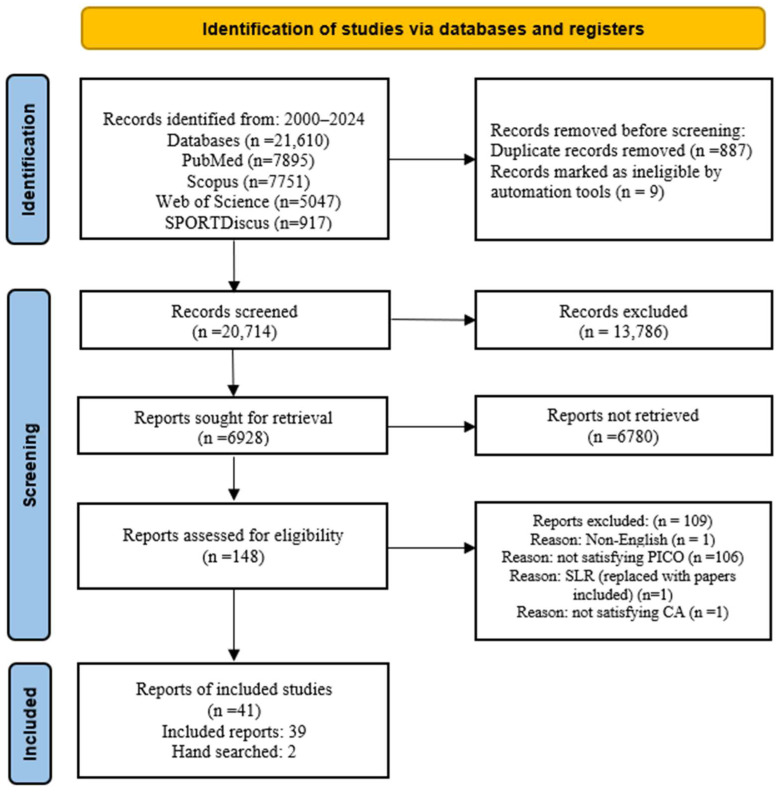
PRISMA flow diagram.

A total of 21,610 records were screened for eligibility, and the full text of 148 reports was retrieved for detailed evaluation. After a comprehensive review of the full texts, a total of forty-one articles met the inclusion criteria after full text review. However, one of the eligible full-text SLRs was replaced by two hand-searched full text articles included in that specific SLR. The identification, screening, and inclusion of the eligibility criteria are presented in the PRISMA flow diagram [23] shown in Figure 1.

### 3.1. Characteristics of the Included Studies

The characteristics of the included studies are summarized in Table 2 and Table 3, which include the study design, participants, outcome measures, main findings, whether significant sex-based variation is reported, and the RoBs of the study. A total of 12,382 individuals participated in the selected studies (n = 7021 males; n = 5361 females). The studies consisted of cohort studies (prospective, retrospective, observational, longitudinal), cross-sectional studies, and case–control studies.

The selected studies were categorized under the following subcategories: sports-related concussion (SRC), military-related concussion, domestic violence, (Table 2) and unclassified category (UC) (Table 3). The studies included in the unclassified category represented the patients admitted to the emergency departments due to falls, road accidents, workplace-related accidents, etc. The study outcomes are synthesised below under the following identified areas: brain image variations, cognitive function variations, gait and balance deformities, variations in electrophysiological measures, and neurosensory alterations.

### 3.2. Sex-Based Variations in Brain Structure Following an mTBI (Neuroimage-Based Findings)

Ten studies investigated sex-based variations in brain images after mTBI. The imaging techniques employed in these studies included Diffusion-Tensor Imaging (DTI), Diffusion-Weighted Magnetic Resonance Imaging (dMRI), MRI (Magnetic Resonance Images), and Resting-state functional Magnetic Resonance Imaging (rs-fMRI). These imaging techniques were utilized to observe the cerebral blood flow (CBF), white matter microstructure, white matter fractional anisotropy (FA), mean diffusivity, cortical thickness, etc. These indices can be examined using imaging techniques. The brain physiology indices of white matter microstructure and CBF demonstrate substantial sex-based differences, where 30% of the white matter regions of interest show absolute sex differences [62], while females are more sensitive to CBF changes [63].

Four out of ten brain image-based studies were under the SRC category representing soccer, ice hockey, hockey, volleyball, football, rugby, basketball, and lacrosse players (Table 2). These studies investigated the sex-based variations in white matter microstructural changes in the brain tissues [31,32,36,37]. Rubin et al.’s study [31] found that women exhibit higher microstructural white matter alteration than men following repetitive sub-concussive head impacts in amateur soccer. Their sample included the players of more than 5 years of amateur-level playing (Table 2). These repetitive head impacts may lead to higher white matter microstructural changes. The reason for these brain microstructural variations between males and females may be observed due to the variations in the heading locations and the biomechanical approaches of force and acceleration experienced during the impact. The heading exposure was associated with lower Fractional Anisotropy (FA) in genu and splenium of the corpus callosum and the pons regions in males, while in females FA-lowering regions include the genu of the corpus callosum; left occipital, right parietal, and right orbitofrontal white matter; left superior longitudinal fasciculus; right cingulum; and right cerebral peduncle [31]. Sollmann et al. [32] also found sex-based differences in structural alterations following exposure to repetitive sub-concussive head impacts in collegiate ice hockey players. Their study observed that increases were observed in pre- vs. post-seasonal absolute difference in FA (0.0268), mean diffusivity (0.0002), axial diffusivity (0.00008), and radial diffusivity (0.00005) in female athletes compared to their male counterparts, showcasing the structural alterations in brain tissue following exposure to repetitive head impacts (Table 2). Churchill et al.’s [36] study, based on university-level athletes (soccer, hockey, volleyball, football, rugby, basketball, and lacrosse players), found that male athletes had greater reductions in occipital-parietal CBF and increases in callosal mean diffusivity, with the greatest effects at one year after the return to play compared to the female athletes. Furthermore, female athletes exhibited reductions in FA of the corona radiata. These variations were observed mainly due to the sex-based differences in white matter microstructure alterations and CBF distribution. Similarly to Rubin’s et al. [31] study on soccer players, Sollmann et al.’s [32] study on ice hockey players also exhibited alterations in the FA in female athletes compared to their male counterparts (Table 2). However, Wright et al.’s [37] study also showed that male concussed athletes (amateur footballers) exhibited significantly greater white matter disruption compared to female concussed athletes following 48 h and two weeks post-injury. Their study on whole-brain voxel analyses found that male athletes demonstrate an increased fibre density in the cingulum compared to their female counterparts following an SRC at 40 h and 2 weeks’ time intervals. Cognitive performance is associated with white matter microstructure and fibre density in the cingulum; therefore, disruptions in these parameters may be linked to early-stage neurodegenerative disorders (Table 2). In addition, Valera et al. [43] investigated brain-imaging-based variations in repetitive mTBIs in the context of domestic violence, finding they may lead to measurable white matter abnormalities especially in posterior and superior corona radiate regions of the affected women’s brain. Both posterior and superior regions of corona radiate serve as major convergence points for long fibre tracts in cerebral cortex to subcortical areas. The damage caused to these regions may impact the working memory, learning attention, and visuospatial and linguistic abilities of the affected person (Table 2). These image-based results indicate that microstructural variations in male and female brain tissue exhibit differences after a head impact, especially with respect to FA variations in white matter and CBF variations. These differences may have a correlation to the structural and anatomical differences in the male and female brains as discussed above, in addition to the level of sex hormones, resulting in differences in cognitive, neurosensory, electrophysiological, and gait and balance deformities. To understand these alterations in treatment interventions, it is essential to understand the sex-based variations in brain tissue microstructures in detail.

In the unclassified category (Table 3), Fakhran et al. [48] found that female patients with mTBI have more extensive white matter abnormalities than male patients, where male patients had significantly decreased fractional anisotropy values in the uncinate fasciculus, which connects the temporal lobe with the frontal lobe with a white matter pathway (mean FA, for males 0.425; 95% confidence interval: 0.375, 0.476 for females 0.443; 95% confidence interval: 0.393, 0.493 with a *p* value > 0.05, (compared with male patients with mTBI) and the Cohen d effect size of 0.720) (Table 3). This detail can be a promising predictor for the persistence of post-concussion symptoms. Shao et al. [50] examined the MRI-based cortical thickness and compared it with neurocognitive outcomes after an mTBI. Females had thicker cortical thickness in the left caudal anterior cingulate cortex than their male counterparts (*p* = 0.004) and higher scores for neurocognitive assessments. These findings can relate to the management strategies for male and female mTBI patients differently (Table 3). Wang et al. [51] examined the influence of sex differences in abnormal intrinsic functional connectivity after acute mTBI, with distinct patterns observed in females compared to males, especially in the right middle frontal gyrus and its connectivity with other brain regions (Table 3). Another Chinese study [53] found that gender and time following the injury significantly affect cognitive performance. An increased CBF was observed in the male patients compared to the females at the acute phase. In addition, male patients showed a reduced CBF in the left inferior frontal cortex at the subacute phase (one month) following the injury. In contrast, no impairments on neuropsychological performance were observed in female patients in their study. These findings suggested that the regional CBF variations could serve as a potential biomarker to investigate the gender variations in the mTBI pathology and the recovery phase [53].

Hsu et al. [49] conducted a study on working memory functional MRI of patients with mTBI one month and two-and-a-half months after the head injury reported at the emergency departments of three selective hospitals. They investigated that female patients with mTBI demonstrate worse working memory activation patterns (with functional MRIs) compared with male patients, and these differences persist over time (Table 3) These decreases in working memory circuits may result from persistent hypoactivation observed in the females post-injury, and it may play a role in poor working memory post-injury and the delayed recovery phase.

By concluding the above brain-imaging-based variations, the following findings were highlighted: females showed smaller disturbances to the CBF compared to their male counterparts. However, sex-based differences in white matter alterations were observed in different regions across the brain. These differences were mainly attributed to the structural and anatomical variations between male and female brains. Moreover, the variations in sex hormones, especially estrogens, progesterone and testosterone levels, may also contribute to the neuroprotective mechanism following an mTBI. Hence, it is important to consider the individual sex hormonal levels, especially for female patients during their menstrual cycle or menopause period.

### 3.3. Sex-Based Variations in Cognitive Function Alterations Following an mTBI

Twenty-four studies discussed the sex-based variations in cognitive functions after mTBI. The assessment tools employed were Immediate Post-Concussion Assessment and Cognitive Testing (ImPACT), Sport Concussion Assessment Tool (SCAT), Standardized Assessment of Concussion (SAC), Post-Concussion Symptom (PCS) scores, Neurobehavioral Symptom Inventory (NSI), Posttraumatic Stress Disorder Checklist (PTSD) checklist, Rivermead Post-Concussion Symptoms Questionnaire (RPQ), computerized Concussion Resolution Indices (CRI) Cambridge Neuropsychological Test Automated Battery, Beck Anxiety Inventory (BAI), Beck Depression Inventory (BDI), Conversational discourse: Canadian Adult Achievement Test (CAAT), Glasgow Outcome Scale Extended (GOSE), and the Test de Rendement pour Francophones (TRF).

The alteration in cognitive functions after an mTBI is one of the commonly observed symptoms in assessing injury severity. Patient-specific factors play a significant role in these symptoms, both immediately after the injury and several months afterward. Sex is considered one of the most important patient-specific factors in the scientific findings, and females show the worst symptoms compared to male patients.

The following findings were highlighted in the SRC subcategory (Table 2). Hutchison et al. [29] found that female university athletes (representing football, soccer, rugby, hockey, basketball, volleyball, lacrosse, and baseball) reported higher baseline SCAT3 symptom scores compared to male athletes (*p* = 0.009). Hamer et al.’s [3] (Table 2) study suggests that a history of concussion (HOC) may have sex-specific effects on CBF, with only males with HOC showing lower CBF relative to controls, but females with multiple concussions showing lower CBF than those with a single concussion. These findings suggest greater overall variability in the effects of HOC on cerebrovascular function for female athletes compared to their male counterparts resulting in a negative impact on cognitive abilities. A Swedish study by Vedung et al. [35] (Table 2) found that compared to male soccer players, female soccer players had worse initial symptom severity scores. These observations may represent the biological differences in male and female neck muscle strength in addition to the hormonal fluctuations. In addition, a recent SRC study by D’Alonzo et al. [40] (Table 2) found that female athletes (participated in basketball, field hockey, football, ice hockey, lacrosse, rugby, soccer, sprint football, water polo, wrestling, baseball, cheerleading, diving, equestrian, fencing, golf, gymnastics, polo, rowing/crew, sailing, skiing, softball, squash, swimming, tennis, track and field/cross country, and volleyball) may experience a longer symptom duration and recovery to sport compared to male athletes, even after controlling for baseline differences and injury characteristics of symptom resolutions, concussion history and academic accommodations [40]. In addition to the differences in neck muscle strength and hormonal fluctuations, females are more vulnerable for the pre-existing psychological conditions. These factors may play a major role in these observations.

A military-based concussion incidence study by Brickell et al. [4] suggests that female service members report more post-concussive symptoms than male service members after mTBI. Their study investigated the patients using the PTSD checklist and NSI. These symptoms include poor concentration, trouble remembering a stressful event, disturbing memories, thoughts, and images with higher PTSD scores (Table 2). These observations may have a relationship with females’ vulnerability in pre-existing psychological conditions and the psycho-social differences experienced in their workplace.

In the unclassified category, five studies found that female sex is independently associated with worse outcome after mTBI at 3 to 6 months, as evidenced by higher PCS scores compared to their male counterparts [44,45,55,58,59] (Table 3). In addition, these findings further encourage the scientific community to assess the mTBI symptom outcomes such as sleep quality, fatigue, phycological status, etc., together with biological sex to understand the severity and the treatment interventions. Wang et al. [52] study suggests that sex differences influence the patterns of abnormal intrinsic functional connectivity after acute mTBI, with distinct patterns observed in females compared to males, particularly about the right middle frontal gyrus and its connectivity with other brain regions, and this may underpin sex-specific clinical symptoms like BAI and BDI (Table 3). LeBlanc et al.’s [56] study also observed that sex-based variations in mTBI based on conversational discourse and reading comprehension. Females performed better than males on letter-category naming. However, for most other cognitive-communication measures presented, males and females performed similarly in the acute stage after a complicated mTBI. In addition, Levin et al. [16] study on posttraumatic stress disorder in mTBI, women exhibited higher levels of posttraumatic stress, depressive, anxiety, and somatization symptoms across all post-injury time points compared with men (Table 3).

Biologically, females report higher levels of physiological stress compared to males [20]. This could show differences in self-reporting symptoms and the pain levels after an mTBI by male and female individuals. Five out of thirteen studies included in the unclassified mTBI category discussed the sex-based variations in self-reported cognitive complaints, and the following findings were highlighted. Levy et al.’s [2] study observed that female participants reported significantly higher levels of cognitive complaint (CCAMCHI scores) compared to males, even after accounting for psychological distress and greater symptom burden (Table 3). However, Anderson et al. [58] study found that sex did not significantly predict CCAMCHI (subjective cognitive impairment), even though female sex was associated with an increased likelihood of PCS reporting. Wågberg et al.’s [60] study observed a worse GOSE outcome in women compared to men (Table 3). Similarly, Losoi et al. [61] study found sex was not associated with chronic post-concussion symptoms after evaluating many neuropsychological indices (Table 3).

Furthermore, Mondello et al. [34] investigated the impact of total tau, neurofilament light chain, glial fibrillary acidic protein, and ubiquitin C-terminal hydrolase-L1 on blood biomarkers after an mTBI (Table 2). Plasma tau levels were significantly higher in females 6 h and 3 days after a concussive event compared to males (10.78 vs. 5.42 pg/mL, *p* = 0.017, and 6.78 vs. 3.49 pg/mL, *p* = 0.0006, respectively), and the relationship between biomarker levels and behavioural outcomes is more evident in females than males.

Lannsjö et al.’s [47] study investigated the computer tomography outcome of an mTBI after 3 months, together with the (RPQ) and GOSE, and reported that female sex is associated with poor outcome, reporting more post-injury symptoms (Table 3). They further emphasized the importance of incorporating sensitive imaging technologies to identify brain pathology together with symptoms, thereby facilitating effective treatment interventions. However, three studies out of 24 included in the cognitive function category have reported no significant sex-based differences (*p* < 0.05) in SAC scores [26], SCAT5 symptom scores [38], computerised CRI [26], and ImPACT battery [42] based on their study populations [26,38]. Even though, Walton et al.’s [41] study on athletes who participated in basketball, baseball/softball, cheer/dance, cross country/track and field, swimming and diving, gymnastics, lacrosse, soccer, squash, and tennis found that no significant sex-based differences in times to reaching any clinical milestone such as time to diagnosis, symptom resolution and RTP (Table 2) Similarly, Sicard et al. [27] analysed sex-based variations in four core cognitive domains: processing speed (Detection Task), attention/vigilance (Identification Task), visual learning (One Card Learning Task) and executive function (1-Back condition of the N-back), where they found no significant sex differences in cognitive tasks except N-back Task (Executive Functions). In the N-back Task, female athletes with a history of concussion responded significantly slower than their male counterparts. The N-back task is more cognitively challenging than the 1-back task; hence, these observations could indicate the persistent cognitive challenges that females exhibit during the chronic phase of the injury. In contrast, females scored significantly higher (*p* = 0.048) on a visual memory test based on pattern recognition compared to their male counterparts [46]. This finding is different from the other cognitive functional variations observed between males and females. Better processing speed in female participants may be linked to their higher education level compared to the male participants in the study cohort.

### 3.4. Sex Based Variations in Gait and Balance Deformities Following an mTBI

Three of the included studies investigated sex-based variations in gait and balance deformities after an mTBI. Females tended to have longer recovery times for gait deformities and return to play/work compared to the male. The following findings were highlighted in the SRC subcategory; Messerschmidt et al. [28] presented their findings based on dual-task cost (DTC) of gait speed, cadence, step length, and step width of the collegiate athletes (Table 2). Their findings show that females demonstrated significantly higher DTC of gait speed cadence, percentage of swing phase, and double-support phase. Howell et al. [33] found that female collegiate athletes (participated in soccer, field hockey, rugby, basketball, ice hockey, softball, and volleyball) take longer to recover objective gait measures of single-task, dual-task, and height-adjusted gait speed after concussion compared to male collegiate athletes (participated in football, baseball, wrestling, ice hockey, lacrosse, sailing) (Table 2).

In the unclassified subcategory, Studenka et al.’s [54] study found that concussed females may be more susceptible to lasting alterations in visual-motor performance than males, especially with increasing numbers of concussion (*p* = 0.031) (Table 3).

### 3.5. Sex-Based Variations in Electrophysiological Measures Following an mTBI

Two studies, by Pauhl et al. [57] and Carrier-Toutant et al. [30], in the included articles discussed the sex-based variations in electrophysiological measures after an mTBI. Pauhl et al. [57] found no significant differences in motor-evoked potential amplitude or cortical silent period duration between males and females following concussion, indicating a lack of differences in these neurophysiological measures between sexes after concussion (Table 3). Carrier-Toutant et al.’s [30] study investigated the early event-related brain potentials measures of P1 and N1 (Table 2). They observed that males exhibited significantly smaller P1 (*p* = 0.08) responses to Emotional Facial Expression stimuli than females. Also, male athletes showed smaller amplitude in N1 component following a concussion compared to female athletes. These findings help to understand the differences in neurophysiological mechanisms related to emotional stimuli for male and female patients with mTBI.

### 3.6. Sex-Based Variations in Neurosensory Alterations Following an mTBI

Lumba-Brown et al. [7] was one of the two included studies that discussed the sex-based variations in neurosensory alterations after mTBI (Table 2). Their study investigated the neurosensory measures of oculomotor impairment, auditory changes, and vestibular impairment. They found that females have a higher incidence of abnormalities in smooth pursuit, convergence, and visual motion sensitivity. Furthermore, males reported higher rates of light sensitivity, while females reported higher rates of noise sensitivity, but these differences were not statistically significant. Caccese et al.’s [39] study reported that female participants scored higher vestibular ocular motor screen total symptom scores at 24–48 h post-injury (*p* = 0.005) compared to their male counterparts (Table 2). Neurosensory variations are widely observed in the post-concussion stage. Understanding the sex-based variations in the neurosensory changes can be incorporated to improve the rehabilitation outcomes.

## 4. Discussion

This systematic review synthesised current evidence on sex-related differences in mild traumatic brain injury (mTBI) severity and recovery across sports, military, domestic violence, and civilian settings. Overall, females consistently showed a higher symptom burden, longer recovery times, and greater cognitive and neurosensory impairments compared with males. These findings emphasise the need to consider sex as a biological variable in both research and clinical practice for mTBI management.

Male and female brains differ in total volume, cortical thickness, and white-to-grey matter ratios, which may influence injury biomechanics and post-injury physiology [64]. When controlling the total brain volume, females show greater total cortical grey matter volumes, while males show greater total white matter volumes [65]. Also, females have a greater volume in the corpus callosum, bilateral parietal lobes, left anterior cingulate gyrus, and left caudate nucleus compared to males, while males have a greater volume in the left superior temporal gyrus [65]. These anatomical variations play a key role in responding to mTBI, as they induce structural and physiological disruption in the white matter regions, especially causing DAI [66]. The external mechanical force experienced by the head during an mTBI includes a mechanical deformation across the brain tissues. Wright et al. [37] observed changes in white matter microstructure within 48 h post-injury, showing striking differences in fibre density. While some studies highlight more white matter alterations in females, which correlate with worse symptom outcomes [31,48,51], others focus on repetitive mild traumatic brain injuries (mTBI) due to domestic violence affecting women [43]. Sollmann et al. [32] also note significant structural changes in collegiate ice hockey players after repeated head impacts. These findings emphasize the vital need for tailored approaches in incorporating sex-based structural differences in mTBI injury management and care.

Alterations in cognitive functions following a mild traumatic brain injury (mTBI) are frequently observed and can serve as key indicators for assessing the severity of the injury. Cognitive functions are altered following an mTBI mainly because axons are damaged or destroyed by acceleration/deceleration forces acting upon the axonal bundles and intracranial blood vessels, resulting in white matter damage. Understanding patient-specific factors is essential, as they significantly impact the symptoms experienced and self-reporting both immediately after the injury and for post-injury assessments. Among these factors, sex has been identified as particularly influential, with research indicating that females may experience more pronounced symptoms and longer recovery time compared to their male counterparts in SRC [35,36,40], military-related mTBI [4] and unclassified mTBI reported at the emergency departments [44,45,55,58,59]. Biologically, females report higher levels of physiological stress compared to males [20]. This could show differences in self-reporting symptoms and the pain levels after an mTBI by male and female individuals. This insight underscores the importance of tailored assessment and intervention strategies based on sex differences in post-injury management following an mTBI.

Impairment in balance and gait is another concerning symptom associated with mTBI [9]. mTBI may alter the gait cycle with slowed walking velocity, greater time in double-leg stance support, and less time in single-leg stance support [67]. These gait deformities are the outcomes of underlying pathophysiological processes due to the concussion, resulting in alterations in neurocognitive functions [5]. This SLR also revealed that females tend to have longer recovery times for gait deformities and return to play/work compared to males [28,33,54].

An mTBI has the potential to alter the electrophysiological measures of the brain. Event-related potentials are one of the most used techniques to observe the cerebral/cognitive consequences that occur due to an mTBI. Only two of the studies [30,57] included in this SLR discuss the electrophysiological observations after an mTBI. The event-related brain potentials (P1, N1) of emotional facial expressions study by Carrier-Toutant et al. [30] identified that males exhibited significantly smaller P1 responses to Emotional Facial Expression stimuli and an amplification in the N1 component than their female counterparts. These observations highlighted the sex-based differences in neurophysiological mechanisms after an mTBI incidence. Hence, it is vital to investigate the event-related electrophysiological potentials to understand the underlying cerebral dysfunctions to address the post-concussive symptoms while addressing the sex-based variations for effective management. Sometimes, conventional imaging assessment may fail to diagnose the underlying impact; in such situations, electrophysiological measures can be used as a promising biomarker to further investigate the injury incident to detect the underlying changes in brain function that are responsible for a patient’s symptoms related to cognition, brain function, and neurological conditions. This indicates that incorporating the sex differences in these interventions may improve the outcome of injury management.

Neurosensory alterations are common after a head trauma due to the extreme damage to the brain tissue, diminished metabolism, and the regulation of cerebral blood flow [68]. Female participants experienced more symptoms related to neurosensory alterations; they exhibited worse ImPACT Visual Memory, slower clinical reaction times, and longer King–Devick Total Time across the timelines of 24–48 h post-injury compared to their male counterparts [39]. Also, females had a higher incidence of abnormalities in smooth pursuit, convergence, and visual motion sensitivity [7]. The sex differences in hormonal fluctuations, especially during the menstrual cycle, and the level of neurochemistry (which are crucial for brain repair and regeneration) may contribute to the injury severity and the symptom outcomes. A tailored assessment may be important when the standard clinical assessments fail to identify the neurosensory symptoms related to the injury, especially for women, as they are more vulnerable to physiological stress compared to males.

Research shows that an mTBI experience during the lifetime may have a positive correlation with neurodegenerative incidents in the later stages of life [69,70,71]. Hence, diagnosis of the incident, proper treatment interventions, and recovery play key roles in reducing the future risk of neurodegenerative diseases. According to the research, sex and age are the primary factors influencing symptom identification, diagnosis and post-injury treatments in patients with mTBI [16]. Therefore, understanding the sex-based factors can guide effective treatment interventions and post-injury management. This will eventually reduce the incidence of neurodegenerative diseases among the impacted population. Additionally, understanding the biochemical changes resulting from head impacts and how they correlate with symptom levels is vital for accurate mTBI diagnosis. This knowledge will significantly enhance treatment outcomes.

People become exposed to various accidents/incidents possibly causing head traumas throughout their lives. These head traumas may be neglected at the point of the incident, mainly due to the severity of the other accident-related injuries. The undiagnosed or untreated head traumas may have a positive correlation with the neurodegenerative diseases in their late life. Understanding the patient-specific factors, like sex, will enhance the effective diagnostic or prognostic interventions by reducing the risk factors and healthcare burdens. This SLR concludes that sex difference plays a vital role in symptom reporting, diagnosis, and recovery phase following a mTBI, mainly due to the biological differences in male and female brain structures, in addition to the sex related biochemical variations, especially in hormones and neurochemistry. Thus, incorporating sex-based variations plays a key role in effective diagnostic and treatment interventions and recovery outcomes. Ultimately, this will contribute to achieving sustainable developmental goals (SDG 3.4) by reducing mortality from non-communicable diseases and promoting mental health.

### Limitations and Future Directions

There are a number of limitations to acknowledge. One of the important limitations was that some of the included studies relied on self-reported concussion/mTBI assessments, which may have introduced bias into the overall findings of these studies. This bias could have influenced the pain severity assessments and the reporting. The selected studies included participants aged 18 years old. However, age is one of the main factors that impact the injury severity and the recovery. Hence, incorporating the younger-aged participants could improve the study’s findings. Heterogeneity of the studies is one of the key limitations of this SLR. It challenges the results synthesis process, especially in interventions, outcome measures, and settings. Furthermore, publication lag could be another limitation, as we conducted the search in November 2024, and since then, some new findings could have been published relevant to this SLR.

The differences in the severity and recovery of mTBI based on sex highlight the need for further research in this area. It is essential to gain a clearer understanding of how brain injuries affect males and females differently. Future studies should concentrate on investigating the structural and biochemical transformations that occur following an mTBI in relation to sex, based on neurobiological differences, while incorporating both neuroimaging and neuropsychological biomarkers. In addition, future studies should focus on female patients during their menstrual cycle or menopause period, as the sex hormone fluctuations vary differently during these periods of their lives. Moreover, the identification and incorporation of the influences of sex chromosome-linked genes may be crucial in injury severity and recovery; therefore, it is vital to explore these gene influences in future research. The inclusion of vulnerable populations such as geriatric, pediatric (children and adolescents), and athletes in the study cohort is crucial when tailoring the early treatment interventions, as it creates a provision to address the variations in individual anatomical and hormonal levels. As stress levels can highly influence the mTBI injury severity and recovery, incorporating the lifestyle and pre-existing mental conditions into the diagnostic stage could improve the treatment outcome. In addition to male and female genders, more research should be focused on understanding the anatomical and biochemical variations in individuals in the nonbinary category. Furthermore, the clinical research should include larger, diverse samples with longitudinal studies to map the symptom trajectories with combined clinical, anatomical (images), genetic, and hormonal data to obtain a clear picture of the mTBI symptoms and recovery stages. This information could be further implemented into risk prediction models to incorporate personalized treatment interventions and better recovery outcomes.

## 5. Conclusions

Mild traumatic brain injuries contribute to more than 90% of reported traumatic brain injuries, which leads to the injury-related long-term consequences, causing higher healthcare costs. This SLR concludes that sex difference plays a vital role in symptom reporting, diagnosis, and the recovery phase following an mTBI. These differences are primarily due to the distinct biological characteristics of male and female brain structures, along with the variations in sex-related biochemistry, especially regarding hormones and neurochemistry. Thus, incorporating sex-based variations plays a key role in effective diagnostic and treatment interventions and recovery outcomes. Therefore, further research should focus more on gaining a clearer understanding of how brain injuries affect males and females differently, especially concerning structural and biochemical variations that result from mTBI.

## Figures and Tables

**Table 1:** Literature search terms, synonyms and related terms.

Search Categories	Injury Mechanism	Severity	Sex/Gender
synonyms/related terms	Sports Related Concussion, Military/blast related, Fall/accidents/head accelerations (whiplash)/trauma, collision, Domestic Violence	Mild traumatic brain injury, Concussion, mTBI	Male, Female

**Table 2:** Overall study characteristics of classified mTBI (SRC, Military, and Domestic violence).

Author	Study Design	Setting	Participants	Outcome Measure	Findings	ROB (%)	Significant Difference Observed?
Zimmer et al. 2013 [26] (USA)	cross-sectional: neuropsychology	SRC	437 NCAA Division II student-athletes (273 males and 164 females) (during a season)	The computerized CRI. The Standardized Assessment of SAC. BESS	No significant sex differences were found with SAC scores, CRIs and BESS score	100	No
Sicard et al. 2018 [27] (Canada and USA)	cross-sectional: neuropsychology	SRC	Asymptomatic student-athletes (98 with a history of concussion (49 male, 49 female) (≥6 months) and 98 matched controls (49 male and 49 female)	Cogstate battery Processing speed (Detection Task) Attention/vigilance (Identification Task) Visual learning (One Card Learning Task) Executive function (1-Back condition of the N-back Task)	N-back Task: Female athletes with a HOC responded significantly slower than their male counterparts. Other Cognitive Tasks: Analyses failed to reveal any significant sex differences	100	Yes No
Messerschmidt et al. 2021 [28] (USA)	cross-sectional: neuropsychology	SRC	98 NCAA Division I student-athletes. 54 male and 44 female (27 with HOC (≥6 months), 58 reported at least one symptom)	DTC of gait speed, cadence, step length, and step width. Percentage of swing and double-support phases. Symptom score and total symptom severity score.	Females demonstrated significantly higher DTC of gait speed cadence, percentage of swing phase, and percentage of double-support phase. Step length and width DTC were not significantly different between female and male athletes. The effect sizes for DTC of gait speed and cadence were moderate for females versus males	100	Yes No Moderate
Hutchison et al. 2017 [29] (Canada)	prospective longitudinal observational study: neuropsychology	SRC	26 University athletes who sustained a sport-related concussion 16 male and 10 female (acute) 26 Control group: 16 male and 10 female	RPQ Physiological Markers: (Cortisol levels and heart rate variability Sport Anxiety Scale Profile of Mood States Perceived Stress Scale Stanford Sleepiness Scale	Female athletes reported higher baseline Sport Concussion SCAT3 symptom scores compared to male athletes (*p* = 0.009). No significant sex differences in the physiological markers	90	Yes No
Carrier-Toutant et al. 2018 [30] (Canada)	Cross-sectional: neuropsychology	SRC	Active athletes 43 Concussion Groups (≥3 months): 21 male and 22 female athletes 40 Control Groups: 20 male and 20 female athletes	Behavioural Measures: Reaction times and accuracy scores from the Emotional Facial Expression identification task. Electrophysiological Measures: Early event-related brain potentials measures (P1 and N1) Clinical Questionnaires: BDI-II, BAI, PCSS, Concussion history questionnaire	Male athletes achieve suppression of the N1 component amplitude after concussion. Neurophysiological mechanisms underlying the processing of emotional stimuli are distinctively affected across sexes after repeated concussions.	95	Yes Yes
Rubin et al. 2018 [31] (USA)	prospective cross-sectional study: neuroimaging	SRC	98 amateur soccer players 49 male and 49 female (≤12 months)	White matter microstructure: DTI, FA demographic characteristics, handedness, and history of concussions. Self-reported soccer heading exposure over the previous 12 months	Women experience more prior concussions and may be more sensitive to the effects of heading at the level of tissue microstructure.	95	Yes
Sollmann et al. 2018 [32] (Canada)	prospective study: neuroimaging and neuropsychology	SRC	25 Collegiate ice hockey players 14 males and 11 females (pre and postseason)	dMRI: FA, mean diffusivity, axial diffusivity, radial diffusivity neurocognitive performance: ImPACT	Sex differences exist in structural alterations following exposure to repetitive sub concussive head impacts	82	Yes
Hamer et al. 2020 [3] (Canada)	cross-sectional: neuroimaging and neuropsychology	SRC	Varsity athletes 56 Concussed (≥1 year): Male 28 and female 28 66 Control: Male 33 and female 33	MRI SCAT3, BESS, SAC, Automatic, Neuropsychological Assessment Metrics (HOC, time since last concussion, and recovery time), BESS	Greater overall variability in the effects of HOC on cerebrovascular function for female athletes	73	Yes
Howell et al. 2020 [33] (USA)	observational cohort: neuropsychology	SRC	94 Collegiate athletes diagnosed with a concussion (≤7 days post-injury, 1.5 and 3.5 months post-injury) 47 males, 47 females	Single-task gait speed Dual-task gait speed Height-adjusted gait speed Post-Concussion Symptom Scale, presence of loss of consciousness, and amnesia.	Female collegiate athletes take longer to recover objective gait measures after concussion compared to male collegiate athletes	86	Yes
Lumba-Brown et al. 2020 [7] (USA)	Retrospective: neuropsychology	SRC	177 Collegiate varsity athletes (103 male and 74 female) (≤72 h post-injury)	Symptom reports. Measures of oculomotor impairment. Auditory changes SCAT5 and Vestibular/Ocular-Motor Screening	Females had significantly more abnormal smooth pursuit (*p*-value: 0.045), convergence (*p*-value: 0.031), and visual motion sensitivity tests results (*p*-value: 0.023) than males. No sex-based differences in neurosensory alterations when grouped by overall auditory, vestibular, or oculomotor impairments	77	Yes No
Mondello et al. 2020 [34] (USA)	Prospective: neuropsychology	SRC	46 Concussed athletes (≤6 h, 2, 3 and 7 days post-injury) 20 males, 26 females	Biomarker measurements: total tau, neurofilament light chain, glial fibrillary acidic protein, and ubiquitin C-terminal hydrolase-L1 Return to play	The relationship between biomarker levels and behavioural outcomes is more evident in females than males.	86	Yes
Vedung et al. 2020 [35] (Sweden)	Prospective: neuropsychology	SRC	959 Elite soccer players 570 male and 389 female (48 h to 3 months post-injury)	Concussion incidence SCAT Risk factors for concussion Return-to-play duration after concussion. Amnesia from SRC, unconsciousness from SRC, and dizziness/seeing stars Recurrence of symptoms during rehabilitation.	Female players had worse initial symptom severity scores, prolonged recovery and experienced longer return-to-play times The concussion incidence itself did not show sex differences	73	Yes No
Churchill et al. 2021 [36] (Canada)	Longitudinal: neuroimaging	SRC	Athletes 61 Concussed group (1–7 days post-injury, medical clearance to RTP, and 1 year post-RTP) (30 male, 31 female) 167 Controls (80 male, 87 female)	MRI measures: CBF, white matter fractional anisotropy and mean diffusivity. SCAT3 or 5 Clinical outcomes: Acute symptoms and time to return to play	Males had greater reductions in occipital-parietal CBF and increases in callosal mean diffusivity, with the greatest effects at 1 year post-return to play. Females had greater reductions in FA of the corona radiata, with the greatest effects at return to play. Females with mTBI were twice as likely to exceed clinical cut-offs for post-concussive and PTSD symptoms, and greatest impact on time-related work demands.	73	Yes Yes Yes
Wright et al. 2021 [37] (Australia)	Observational: Neuroimaging	SRC	Athletes 14 Concussed Group (after 24–48 h and 2 weeks post-injury): 8 males, 6 females 16 Control Group: 9 males, 7 females	dMRI Self-reported symptoms	Male athletes reported more symptoms and greater symptom severity. Male concussed athletes exhibiting significantly greater white matter disruption.	86	Yes Yes
Teramoto et al. 2022 [38] (USA)	Retrospective: neuropsychology	SRC	111 College athletes who sustained concussions (24–72 h post-injury) 59 male and 52 female	SCAT5, SAC, and BESS. Vestibular Oculomotor Screening	No sex differences in common concussion measures	100	No
Caccese et al. 2024 [39] (USA)	Prospective: neuropsychology	SRC	906 NCAA 350 male, 556 female (6 h, 24–48 h (RTP), 6 months post-injury)	SAC, BESS, Brief Symptom Inventory-18, ImPACT, SCAT-3 symptom evaluation Clinical Reaction Time King-Devick test Vestibular Ocular Motor Screen 12-item Short-Form Health Hospital Anxiety and Depression Scale Satisfaction with Life Scale	Female athletes exhibit a greater symptom burden, their overall recovery trajectories for most assessments are similar to male athletes	100	Yes
D’Alonzo et al. 2024 [40] (USA)	Prospective: neuropsychology	SRC	1160 Sport-related concussed patients (during the season) 667 male 493 female	Symptom Resolution Return to Sport Return to Activity: Academic Accommodation Resolution	Female athletes experience a longer symptom duration and recovery to sport. The sport contact level (contact vs. non-contact) did not significantly modify any of the sex-outcome associations.	77	Yes No
Walton et al. 2024 [41] (USA)	Retrospective: neuropsychology	SRC	196 Collegiate athletes (during the season) 57 male and 139 female	Time to diagnosis Symptom Resolution Unrestricted Return-to-Sport	No significant differences in times to reaching any clinical milestone by sex, athletic trainer exposure, or their interaction	77	No
Covassin et al., 2007 [42] (USA)	Prospective: neuropsychology	SRC	79 Concussed collegiate athletes (preseason and, 2–8 days postinjury) 41 male and 38 female	ImPACT Endorsed post-concussion symptoms	No main effect of sex.	77	No Yes
Brickell et al. 2017 [4] (USA)	Cohort: neuropsychology	Military	172 United States military service members who had sustained from mTBI (≤24 months post-injury). 86 male, 86 female	Neurobehavioral Symptom Inventory PTSD Checklist	No sex differences across all demographic and injury-related variables Female service members report more post-concussive symptoms particularly in the affective, somatic, and vestibular domains	95	No Yes
Valera et al. 2019 [43] (USA)	Retrospective: neuroimaging and neuropsychology	Domestic Violence	20 women with histories of intimate partner violence (chronic)	White Matter Microstructure: Measured using dMRI. Cognitive Measures: Learning, Memory, cognitive Flexibility Intimate partner violence-related mTBI Score: A score developed for the study reflecting the number and recency of intimate partner violence related mTBIs.	Repetitive mTBIs may lead to measurable white matter abnormalities in women.	64	Yes

Note: SRC—Sports Related Concussion, ROB: Risk of Bias, NCAA: National Collegiate Athletic Association, CRI: Concussion Resolution Indices, SAC: Standardized Assessment of Concussion; BESS: Balance Error Scoring System, HOC: History of Concussion, DTC: Dual-Task Cost, SCAT3/5: Sport Concussion Assessment Tool 3/5, P1 and N1: brain potentials measures, BDI-II: Beck Depression Inventory II, BAI: Beck Anxiety Inventory, PCSS: Post-Concussion Symptoms Scale, DTI: Diffusion-Tensor Imaging, FA: Fractional Anisotropy, dMRI: Diffusion-Weighted Magnetic Resonance Imaging, ImPACT: Immediate Post-Concussion Assessment and Cognitive Testing, MRI: Magnetic Resonance Imaging, CBF: Cerebral Blood Flow, mTBI: Mild Traumatic Brain Injury, PTSD: Posttraumatic Stress Disorder Checklist.

**Table 3:** Overall study characteristics of unclassified mTBI (Based on emergency department records).

Author	Study Design	Setting	Participants	Outcome Measure	Findings	ROB (%)	Significant Difference Observed?
Maria et al. 2001 [44] (USA)	Observational: neuropsychology	UC	Participants 50 mild head injury (chronic) and 48 controls (37 male and 61 female)	General Health Questionnaire-28 PCSC	Females being more variable in their self-reported post-concussion symptomatology Males show more stable scores for PCSC.	77	Yes Yes
Bazarian et al. 2010 [45] (USA)	Cohort: neuropsychology	UC	1425 Patients with mTBI (3 months post-injury): 782 male and 643 females	PCSS Number of days to return to normal activities Number of days of work missed	Females show worse outcome after mTBI at 3 months, as evidenced by higher PCSS and delays in return to activities and work	77	Yes
Moore et al. 2010 [46] (USA)	Observational: neuropsychology	UC	54 Patients with mTBI (≥1 year post-injury): 22 male and 32 female	Cambridge Neuropsychological Test Automated Battery	Females scored significantly higher for visual memory test than males.	73	Yes
Lannsjö et al. 2013 [47] (Sweden)	Cohort: neuroimaging and neuropsychology	UC	1262 Patients with mTBI (≤24 h and 3 months): 751 males and 511 females	CT RPQ GCS GOSE	No key findings related to sex-based variations.	82	No
Fakhran et al. 2014 [48] (USA)	Retrospective: neuroimaging and neuropsychology	UC	69 Patients with mTBI (3 days and 3 months post-injury): 47 male and 22 females 21 control subjects: 10 male and 11 female	White Matter Integrity Neurocognitive Testing Time to Symptom Resolution	Female patients have more extensive white matter abnormalities, and these abnormalities are associated with worse clinical outcomes.	68	Yes
Hsu et al. 2015 [49] (Taiwan)	Prospective: neuroimaging and neuropsychology	UC	30 patients with MTBI (4 weeks and 10 weeks post-injury): 15 male and 15 female 30 control subjects: 15 male and 15 female	MRI Working Memory Brain Activation Neuropsychological Tests	Female patients demonstrate different and more widespread working memory activation patterns, and these differences persist over time	73	Yes
Shao et al. 2018 [50] (China)	Observational: neuroimaging and neuropsychology	UC	32 patients with mTBI (≤24 h): 18 males and 14 females 30 healthy controls: 14 males and 18 females	Cortical Thickness: high-resolution MRI images Neurocognitive Assessments: Trail-Making Test Part A and Digit Symbol coding score, Forward Digit Span and Backward Digit Span, working memory, and executive function, Verbal Fluency Test for language ability, semantic memory, and executive function, PTSD Checklist-Civilian Version	Female patients showed a non-significant tendency of increased cortical thickness in the left insula cortex. Female patients had higher scores on the PTSD Checklist–Civilian	77	Yes Yes
Wang et al. 2018 [51] (China)	Cross-sectional: neuroimaging and neuropsychology	UC	54 Patients with mTBI (≤7 days post-injury): 27 males, 27 females 34 healthy control subjects: 17 males, 17 females	rs-fMRI Neuropsychological Assessments: Trail-Making Test Part A Digit Symbol Coding score, Forward Digit Span. Backward Digit Span, Verbal Fluency Test, semantic memory, and executive function PTSD Checklist–Civilian Version	A distinct pattern was observed in the right middle frontal gyrus and its connectivity with other brain regions in females, which may explain sex-specific clinical symptoms such as PTSD.	82	Yes
Wang et al. 2018 [52] (Taiwan)	Observational: neuropsychology	UC	192 Patients with mTBI (1 and 6 weeks post-injury): 61 male and 131 female	Self-reporting questionnaires: BAI BDI Blood samples	Male patients carrying T allele of rs6265 had significantly higher BAI scores in the first week (*p* = 0.01) and the sixth week (*p* = 0.038). They also showed higher BDI scores in the first week (*p* = 0.029) and the sixth week (*p* = 0.021)	68	Yes
					Female patients carrying the T allele had significantly higher BDI scores in the first week (*p* = 0.015), but this difference was not observed in the sixth week.		Yes
Bai et al. 2019 [53] (China)	Longitudinal: neuroimaging and neuropsychology	UC	41 Patients with mTBI (acute stage to 1 month post-injury): 25 male, 16 female 25 Healthy controls: 11 male, 14 female	Neuropsychological Tests: Rivermead Post-Concussion Symptom Questionnaire and PTSD Checklist civilian CBF (MRI)	Gender and the time following the injury (sub-acute phase) has a significant effect on the CBF in frontal cortex A reduced CBF observed in male mTBI patients correlated with poorer cognitive performance in subacute phase compared to the acute phase, a correlation not seen in female patients.	77	Yes Yes
Studenka et al. 2019 [54] (USA)	Cross-sectional: neuropsychology	UC	73 Concussion group (≥6 months post-injury): 51 males, 22 females 75 control group: 49 males, 26 females	seated visual-motor tracking task	Concussed females may be more susceptible to lasting alterations in visual-motor performance with increasing numbers of concussions	64	Yes
Yue et al. 2019 [55] (USA)	Prospective: neuroimaging and neuropsychology	UC	100 Patients with mTBI (6 months post-injury): 71 males and 29 females.	GOSE PCSS/PTSD RPQBrief Symptom Inventory Satisfaction with Life Scale Trail Making Test Wechsler Adult Intelligence Scale Fourth Edition, Processing Speed Index California Verbal Learning Test, Second Edition	Females had a significantly higher proportion of unfavourable GOSE outcomes, PCSS, lower quality of life, higher rates of clinically significant depression, and worse sleep quality at six months.	77	Yes
LeBlanc et al. 2021 [56] (Canada)	Retrospective: neuroimaging and neuropsychology	UC	128 Patients with mTBI (acute): 84 male and 44 female	CT scan Auditory comprehension: Boston Diagnostic Aphasia Examination. Verbal reasoning: Detroit Test of Learning Aptitude. Confrontation naming: Boston Naming Test Verbal fluency Conversational discourse: Protocole Montréal d’évaluation de la communication. Reading comprehension: (CAAT) and (TRF)	Females performed better on letter-category naming. Other cognitive-communication measures: males and females performed similarly in the acute stage	77	Yes
Levin et al. 2021 [16] (USA)	Prospective: neuropsychology	UC	2000 Patients with mTBI (2 weeks, 3 months, 6 months, and 12 months post-injury): 1331 male and 669 female Control: 299 patients with orthopaedic trauma: 199 male and 100	RPQ PTSD Checklist Patient Health Questionnaire Brief Symptom Inventory	Females exhibited higher levels of post-traumatic stress, depressive, anxiety, and somatization symptoms across all post-injury time points compared with males.	82	Yes
Pauhl et al. 2022 [57] (USA)	Observational: neuropsychology	UC	21 Patients with concussion (within 72 h, 1 week, and 2 weeks post-injury): 12 males and 9 females 21 controls: 12 males and 9 females	Motor evoked potential amplitude: to assess corticospinal excitability. Cortical silent period duration: to assess corticospinal inhibition. Electromyography: to record evoked potentials from the first dorsal interosseous muscle	Males had significantly longer Cortical Silent Period durations and this indicates greater corticospinal inhibition in males. No significant differences in motor-evoked potential amplitude or cortical silent period duration between males	91	Yes No
Anderson et al. 2023 [58] (Australia)	Retrospective: neuropsychology	UC	94 Patients with mTBI (1–4 days and 6–12 weeks post-injury): 71 males, 23 females	RPQ CCAMCHI Inventory of Depressive Symptomatology for depression, BAI for anxiety, PTSD Checklist for PTSD symptoms.	Female sex was associated with an increased likelihood of PCS reporting. Sex did not significantly predict CCAMCHI (subjective cognitive impairment)	82	Yes No
Anderson et al. 2023 [59] (Australia)	Prospective: neuropsychology	UC	92 Patients with mTBI (6–12 weeks post-injury): 69 males and 23 females	RPQ, Pittsburgh Sleep Quality Index, Multidimensional Fatigue Inventory, Psychological distress: (inventory of depressive symptomatology–self-report, BAI, and PTSD Checklist	Female sex is a significant predictor of PCS, even when accounting for the influence of fatigue and sleep disturbance and symptom reporting.	82	Yes
Wågberg et al. 2023 [60] (Sweden)	Prospective: neuropsychology	UC	595 Patients with mTBI (7–8 years post-injury): male 328 and female 267	RPQ GOSE	The symptom burden was higher in females and presented a worse GOSE outcome.	91	Yes
Levy et al. 2024 [2] (Australia)	Prospective: neuropsychology	UC	68 Patients with mTBI (6–10 weeks post-injury): 53 males and 15 females 40 Control group (trauma): 33 males and 7 females	RPQ CCAMCHI inventory of depressive symptomatology-self-report (BAI, and PTSD Checklist Short-Form McGill Pain Questionnaire Weschler Test of Adult Reading Symbol Digit Modalities Test Rey Auditory Verbal Learning Test	Female participants reported significantly higher levels of cognitive complaint, even after accounting for psychological distress and greater symptom burden.	77	Yes
Losoi et al. 2016 [61] (Finland)	Prospective: neuropsychology	UC	74 Patients with mTBI (1, 6 and 12 months post-injury): 45 male, 29 female 40 healthy controls: 20 male, 20 female	Symptoms: RPQ. GOSE PTSD-Checklist-Civilian Version Barrow Neurological Institute Fatigue Scale Insomnia Severity Index The Pain Subscale of the Ruff Neurobehavioral Inventory BDI- Second Edition The Resilience Scale QOLIBRI-OS The Rey Auditory Verbal Learning Test Return to work	Sex was not associated with chronic PCS.	91	No

Note: UC: Unclassified, ROB: Risk of Bias, PCSC: Post-Concussion Symptom Checklist, PCSS: Post-Concussion Symptoms Scale, mTBI: Mild Traumatic Brain Injury, RPQ: Rivermead Post Concussion Symptoms Questionnaire, GCS: Glasgow Coma Scale, MRI: Magnetic Resonance Imaging, PTSD: Posttraumatic Stress Disorder, rs-fMRI: Resting-state functional Magnetic Resonance Imaging, BAI: Beck Anxiety Inventory, BDI: Beck Depression Inventory, CBF: Cerebral Blood Flow, GOSE: Glasgow Outcome Scale Extended, QOLIBRI-OS: Quality of Life after Brain Injury–Overall Scale, CAAT: Canadian Adult Achievement Test, TRF: Test de Rendement pour Francophones, CCAMCHI: Cognitive Complaint After Mild Closed Head Injury.

## Data Availability

No new data were created or analyzed in this study. Data sharing is not applicable to this article.

## Abbreviations

The following abbreviations are used in this manuscript:

BESS	Balance Error Scoring System
BAI	Beck Anxiety Inventory
BDI	Beck Depression Inventory
CAAT	Canadian Adult Achievement Test
CBF	Cerebral Blood Flow
CCAMCHI	Cognitive Complaint After Mild Closed Head Injury
CT	Computer Tomography
CRI	Concussion Resolution Indices
DAI	Diffuse Axonal Injury
DTI	Diffusion-Tensor Imaging
dMRI	Diffusion-Weighted Magnetic Resonance Imaging
DTC	Dual-Task Cost
FA	Fractional Anisotropy
GCS	Glasgow Coma Scale
GOSE	Glasgow Outcome Scale Extended
HOC	History of Concussion
ImPACT	Immediate Post-Concussion Assessment and Cognitive Testing
mTBI	Mild Traumatic Brain Injury
MRI	Magnetic Resonance Imaging
NA	Not applicable
NSI	Neurobehavioral Symptom Inventory
PICO	Population, Intervention, Comparator, and Outcome
PCSC	Post-Concussion Symptom checklist
PCS	Post-Concussion Symptom
PCSS	Post-Concussion Symptoms Scale
PTSD	Posttraumatic Stress Disorder Checklist
PRISMA	Preferred Reporting Items for Systematic Reviews
QOLIBRI-OS	Quality of Life after Brain Injury-Overall Scale
rs-fMRI	Resting-state functional Magnetic Resonance Imaging
RPQ	Rivermead Post Concussion Symptoms Questionnaire
RoB	Risk of Bias
SAC	Standardized Assessment of Concussion
SCAT	Sport Concussion Assessment Tool
SRC	Sports Related Concussion
SDG	Sustainable Developmental Goals
SLR	Systematic Literature Review
TBI	Traumatic Brain Injury
TRF	Test de Rendement pour Francophones
UC	unclassified category
