# Correction: Arachchi et al. Sex Differences in Severity and Recovery Following Mild Traumatic Brain Injury: A Systematic Review. *Brain Sci.* 2026, *16*, 77

**DOI:** 10.3390/brainsci16040373

**Published:** 2026-03-30

**Authors:** Shanika Arachchi, Ed Daly, Anushree Dwivedi, Lisa Ryan

**Affiliations:** 1 Department of Sport, Exercise and Nutrition, Atlantic Technological University, H91 T8NW Galway, Ireland; shanika.arachchi@research.atu.ie (S.A.); ed.daly@atu.ie (E.D.); 2 Department of Mechanical and Industrial Engineering, Atlantic Technological University, H91 T8NW Galway, Ireland; anushree.dwivedi@atu.ie

## Error in Table

In the original publication [1], there were mistakes in Tables 2 and 3 as published. The total number of participants was not included in the initial version, and the related information is updated in Table 2 and Table 3. Additionally, some of the missing information was also updated. The corrected Table 2 and Table 3 appear below. 

## Text Correction

There were errors in the original publication.

## Abstract—Results section

The total number of participants were updated as follows, “Forty-one studies involving 12,382 participants (7021 males; 5361 females)”.

## 3. Results

### 3.1. Characteristics of the Included Studies

Total number of participants were updated as “A total of 12,382 individuals participated in the selected studies (n = 7021 males; n = 5361 females)”.

### 3.2. Sex-Based Variations in Brain Structure Following an mTBI (Neuroimage-Based Findings) 

Image based study number was updated as “Ten” studies investigated sex-based variations in brain images after mTBI. The same information was updated in the next paragraph as “Four out of ten brain image-based studies were under the SRC category”.

### 3.3. Sex-Based Variations in Cognitive Function Alterations Following an mTBI 

An information reporting error was identified in the 3rd paragraph as “Walton et al.’s [41] study on athletes who participated in football, volleyball, wrestling, rowing, field hockey, and equestrian found that female athletes demonstrated a significantly longer time to symptom resolution, especially related to depression and anxiety (*p* < 0.001) compared to male athletes (Table 2)”. The information reporting error was corrected as follows in Section 3.3 and Section 8th paragrph, “Even though, Walton et al.’s [41] study on athletes who participated in basketball, baseball/softball, cheer/dance, cross country/track and field, swimming and diving, gymnastics, lacrosse, soccer, squash, and tennis found that no significant sex-based differences in times to reaching any clinical milestone such as time to diagnosis, symptom resolution and RTP (Table 2)”.

Another information reporting error was identified in the 5th paragraphs as “Wang et al. [52] study suggests that sex differences influence the patterns of abnormal intrinsic functional connectivity after acute mTBI, with distinct patterns observed in females compared to males, particularly about the right middle frontal gyrus and its connectivity with other brain regions, and this may underpin sex-specific clinical symptoms like PTSD (Table 3).” It was corrected and updated as follows “Wang et al. [52] study suggests that sex differences influence the patterns of abnormal intrinsic functional connectivity after acute mTBI, with distinct patterns observed in females compared to males, particularly about the right middle frontal gyrus and its connectivity with other brain regions, and this may underpin sex-specific clinical symptoms like BAI and BDI (Table 3)”. 

In 6th paragraph, another information reporting error was identified as: “Losoi et al. [61] study found that females reported more symptoms compared to males in the cognitive domain (Table 3).” It was corrected and updated as follows “Losoi et al. [61] study found sex was not associated with chronic post-concussion symptoms after evaluating many neuropsychological indices (Table 3).”

## 4. Discussion

The references were updated in the following sentence in the 4th paragraph “Among these factors, sex has been identified as particularly influential, with research indicating that females may experience more pronounced symptoms and longer recovery time compared to their male counterparts in SRC [35,36,40], military-related mTBI [4] and ….”

Additionally, “(2/42)” was removed from the initial version to improve the sentence.

The authors state that the scientific conclusions are unaffected. This correction was approved by the Academic Editor. The original publication has also been updated.

## Figures and Tables

**Table 2:** Overall study characteristics of classified mTBI (SRC, Military, and Domestic violence).

Author	Study Design	Setting	Participants	Outcome Measure	Findings	ROB (%)	Significant Difference Observed?
Zimmer et al. 2013 [26] (USA)	cross-sectional: neuropsychology	SRC	437 NCAA Division II student-athletes (273 males and 164 females) (during a season)	The computerized CRI. The Standardized Assessment of SAC. BESS	No significant sex differences were found with SAC scores, CRIs and BESS score	100	No
Sicard et al. 2018 [27] (Canada and USA)	cross-sectional: neuropsychology	SRC	Asymptomatic student-athletes (98 with a history of concussion (49 male, 49 female) (≥6 months) and 98 matched controls (49 male and 49 female)	Cogstate battery Processing speed (Detection Task) Attention/vigilance (Identification Task) Visual learning (One Card Learning Task) Executive function (1-Back condition of the N-back Task)	N-back Task: Female athletes with a HOC responded significantly slower than their male counterparts. Other Cognitive Tasks: Analyses failed to reveal any significant sex differences	100	Yes No
Messerschmidt et al. 2021 [28] (USA)	cross-sectional: neuropsychology	SRC	98 NCAA Division I student-athletes. 54 male and 44 female (27 with HOC (≥6 months), 58 reported at least one symptom)	DTC of gait speed, cadence, step length, and step width. Percentage of swing and double-support phases. Symptom score and total symptom severity score.	Females demonstrated significantly higher DTC of gait speed cadence, percentage of swing phase, and percentage of double-support phase. Step length and width DTC were not significantly different between female and male athletes. The effect sizes for DTC of gait speed and cadence were moderate for females versus males	100	Yes No Moderate
Hutchison et al. 2017 [29] (Canada)	prospective longitudinal observational study: neuropsychology	SRC	26 University athletes who sustained a sport-related concussion 16 male and 10 female (acute) 26 Control group: 16 male and 10 female	RPQ Physiological Markers: (Cortisol levels and heart rate variability Sport Anxiety Scale Profile of Mood States Perceived Stress Scale Stanford Sleepiness Scale	Female athletes reported higher baseline Sport Concussion SCAT3 symptom scores compared to male athletes (*p* = 0.009). No significant sex differences in the physiological markers	90	Yes No
Carrier-Toutant et al. 2018 [30] (Canada)	Cross-sectional: neuropsychology	SRC	Active athletes 43 Concussion Groups (≥3 months): 21 male and 22 female athletes 40 Control Groups: 20 male and 20 female athletes	Behavioural Measures: Reaction times and accuracy scores from the Emotional Facial Expression identification task. Electrophysiological Measures: Early event-related brain potentials measures (P1 and N1) Clinical Questionnaires: BDI-II, BAI, PCSS, Concussion history questionnaire	Male athletes achieve suppression of the N1 component amplitude after concussion. Neurophysiological mechanisms underlying the processing of emotional stimuli are distinctively affected across sexes after repeated concussions.	95	Yes Yes
Rubin et al. 2018 [31] (USA)	prospective cross-sectional study: neuroimaging	SRC	98 amateur soccer players 49 male and 49 female (≤12 months)	White matter microstructure: DTI, FA demographic characteristics, handedness, and history of concussions. Self-reported soccer heading exposure over the previous 12 months	Women experience more prior concussions and may be more sensitive to the effects of heading at the level of tissue microstructure.	95	Yes
Sollmann et al. 2018 [32] (Canada)	prospective study: neuroimaging and neuropsychology	SRC	25 Collegiate ice hockey players 14 males and 11 females (pre and postseason)	dMRI: FA, mean diffusivity, axial diffusivity, radial diffusivity neurocognitive performance: ImPACT	Sex differences exist in structural alterations following exposure to repetitive sub concussive head impacts	82	Yes
Hamer et al. 2020 [3] (Canada)	cross-sectional: neuroimaging and neuropsychology	SRC	Varsity athletes 56 Concussed (≥1 year): Male 28 and female 28 66 Control: Male 33 and female 33	MRI SCAT3, BESS, SAC, Automatic, Neuropsychological Assessment Metrics (HOC, time since last concussion, and recovery time), BESS	Greater overall variability in the effects of HOC on cerebrovascular function for female athletes	73	Yes
Howell et al. 2020 [33] (USA)	observational cohort: neuropsychology	SRC	94 Collegiate athletes diagnosed with a concussion (≤7 days post-injury, 1.5 and 3.5 months post-injury) 47 males, 47 females	Single-task gait speed Dual-task gait speed Height-adjusted gait speed Post-Concussion Symptom Scale, presence of loss of consciousness, and amnesia.	Female collegiate athletes take longer to recover objective gait measures after concussion compared to male collegiate athletes	86	Yes
Lumba-Brown et al. 2020 [7] (USA)	Retrospective: neuropsychology	SRC	177 Collegiate varsity athletes (103 male and 74 female) (≤72 h post-injury)	Symptom reports. Measures of oculomotor impairment. Auditory changes SCAT5 and Vestibular/Ocular-Motor Screening	Females had significantly more abnormal smooth pursuit (*p*-value: 0.045), convergence (*p*-value: 0.031), and visual motion sensitivity tests results (*p*-value: 0.023) than males. No sex-based differences in neurosensory alterations when grouped by overall auditory, vestibular, or oculomotor impairments	77	Yes No
Mondello et al. 2020 [34] (USA)	Prospective: neuropsychology	SRC	46 Concussed athletes (≤6 h, 2, 3 and 7 days post-injury) 20 males, 26 females	Biomarker measurements: total tau, neurofilament light chain, glial fibrillary acidic protein, and ubiquitin C-terminal hydrolase-L1 Return to play	The relationship between biomarker levels and behavioural outcomes is more evident in females than males.	86	Yes
Vedung et al. 2020 [35] (Sweden)	Prospective: neuropsychology	SRC	959 Elite soccer players 570 male and 389 female (48 h to 3 months post-injury)	Concussion incidence SCAT Risk factors for concussion Return-to-play duration after concussion. Amnesia from SRC, unconsciousness from SRC, and dizziness/seeing stars Recurrence of symptoms during rehabilitation.	Female players had worse initial symptom severity scores, prolonged recovery and experienced longer return-to-play times The concussion incidence itself did not show sex differences	73	Yes No
Churchill et al. 2021 [36] (Canada)	Longitudinal: neuroimaging	SRC	Athletes 61 Concussed group (1–7 days post-injury, medical clearance to RTP, and 1 year post-RTP) (30 male, 31 female) 167 Controls (80 male, 87 female)	MRI measures: CBF, white matter fractional anisotropy and mean diffusivity. SCAT3 or 5 Clinical outcomes: Acute symptoms and time to return to play	Males had greater reductions in occipital-parietal CBF and increases in callosal mean diffusivity, with the greatest effects at 1 year post-return to play. Females had greater reductions in FA of the corona radiata, with the greatest effects at return to play. Females with mTBI were twice as likely to exceed clinical cut-offs for post-concussive and PTSD symptoms, and greatest impact on time-related work demands.	73	Yes Yes Yes
Wright et al. 2021 [37] (Australia)	Observational: Neuroimaging	SRC	Athletes 14 Concussed Group (after 24–48 h and 2 weeks post-injury): 8 males, 6 females 16 Control Group: 9 males, 7 females	dMRI Self-reported symptoms	Male athletes reported more symptoms and greater symptom severity. Male concussed athletes exhibiting significantly greater white matter disruption.	86	Yes Yes
Teramoto et al. 2022 [38] (USA)	Retrospective: neuropsychology	SRC	111 College athletes who sustained concussions (24–72 h post-injury) 59 male and 52 female	SCAT5, SAC, and BESS. Vestibular Oculomotor Screening	No sex differences in common concussion measures	100	No
Caccese et al. 2024 [39] (USA)	Prospective: neuropsychology	SRC	906 NCAA 350 male, 556 female (6 h, 24–48 h (RTP), 6 months post-injury)	SAC, BESS, Brief Symptom Inventory-18, ImPACT, SCAT-3 symptom evaluation Clinical Reaction Time King-Devick test Vestibular Ocular Motor Screen 12-item Short-Form Health Hospital Anxiety and Depression Scale Satisfaction with Life Scale	Female athletes exhibit a greater symptom burden, their overall recovery trajectories for most assessments are similar to male athletes	100	Yes
D’Alonzo et al. 2024 [40] (USA)	Prospective: neuropsychology	SRC	1160 Sport-related concussed patients (during the season) 667 male 493 female	Symptom Resolution Return to Sport Return to Activity: Academic Accommodation Resolution	Female athletes experience a longer symptom duration and recovery to sport. The sport contact level (contact vs. non-contact) did not significantly modify any of the sex-outcome associations.	77	Yes No
Walton et al. 2024 [41] (USA)	Retrospective: neuropsychology	SRC	196 Collegiate athletes (during the season) 57 male and 139 female	Time to diagnosis Symptom Resolution Unrestricted Return-to-Sport	No significant differences in times to reaching any clinical milestone by sex, athletic trainer exposure, or their interaction	77	No
Covassin et al., 2007 [42] (USA)	Prospective: neuropsychology	SRC	79 Concussed collegiate athletes (preseason and, 2–8 days postinjury) 41 male and 38 female	ImPACT Endorsed post-concussion symptoms	No main effect of sex.	77	No Yes
Brickell et al. 2017 [4] (USA)	Cohort: neuropsychology	Military	172 United States military service members who had sustained from mTBI (≤24 months post-injury). 86 male, 86 female	Neurobehavioral Symptom Inventory PTSD Checklist	No sex differences across all demographic and injury-related variables Female service members report more post-concussive symptoms particularly in the affective, somatic, and vestibular domains	95	No Yes
Valera et al. 2019 [43] (USA)	Retrospective: neuroimaging and neuropsychology	Domestic Violence	20 women with histories of intimate partner violence (chronic)	White Matter Microstructure: Measured using dMRI. Cognitive Measures: Learning, Memory, cognitive Flexibility Intimate partner violence-related mTBI Score: A score developed for the study reflecting the number and recency of intimate partner violence related mTBIs.	Repetitive mTBIs may lead to measurable white matter abnormalities in women.	64	Yes

Note: SRC—Sports Related Concussion, ROB: Risk of Bias, NCAA: National Collegiate Athletic Association, CRI: Concussion Resolution Indices, SAC: Standardized Assessment of Concussion; BESS: Balance Error Scoring System, HOC: History of Concussion, DTC: Dual-Task Cost, SCAT3/5: Sport Concussion Assessment Tool 3/5, P1 and N1: brain potentials measures, BDI-II: Beck Depression Inventory II, BAI: Beck Anxiety Inventory, PCSS: Post-Concussion Symptoms Scale, DTI: Diffusion-Tensor Imaging, FA: Fractional Anisotropy, dMRI: Diffusion-Weighted Magnetic Resonance Imaging, ImPACT: Immediate Post-Concussion Assessment and Cognitive Testing, MRI: Magnetic Resonance Imaging, CBF: Cerebral Blood Flow, mTBI: Mild Traumatic Brain Injury, PTSD: Posttraumatic Stress Disorder Checklist.

**Table 3:** Overall study characteristics of unclassified mTBI (Based on emergency department records).

Author	Study Design	Setting	Participants	Outcome Measure	Findings	ROB (%)	Significant Difference Observed?
Maria et al. 2001 [44] (USA)	Observational: neuropsychology	UC	Participants 50 mild head injury (chronic) and 48 controls (37 male and 61 female)	General Health Questionnaire-28 PCSC	Females being more variable in their self-reported post-concussion symptomatology Males show more stable scores for PCSC.	77	Yes Yes
Bazarian et al. 2010 [45] (USA)	Cohort: neuropsychology	UC	1425 Patients with mTBI (3 months post-injury): 782 male and 643 females	PCSS Number of days to return to normal activities Number of days of work missed	Females show worse outcome after mTBI at 3 months, as evidenced by higher PCSS and delays in return to activities and work	77	Yes
Moore et al. 2010 [46] (USA)	Observational: neuropsychology	UC	54 Patients with mTBI (≥1 year post-injury): 22 male and 32 female	Cambridge Neuropsychological Test Automated Battery	Females scored significantly higher for visual memory test than males.	73	Yes
Lannsjö et al. 2013 [47] (Sweden)	Cohort: neuroimaging and neuropsychology	UC	1262 Patients with mTBI (≤24 h and 3 months): 751 males and 511 females	CT RPQ GCS GOSE	No key findings related to sex-based variations.	82	No
Fakhran et al. 2014 [48] (USA)	Retrospective: neuroimaging and neuropsychology	UC	69 Patients with mTBI (3 days and 3 months post-injury): 47 male and 22 females 21 control subjects: 10 male and 11 female	White Matter Integrity Neurocognitive Testing Time to Symptom Resolution	Female patients have more extensive white matter abnormalities, and these abnormalities are associated with worse clinical outcomes.	68	Yes
Hsu et al. 2015 [49] (Taiwan)	Prospective: neuroimaging and neuropsychology	UC	30 patients with MTBI (4 weeks and 10 weeks post-injury): 15 male and 15 female 30 control subjects: 15 male and 15 female	MRI Working Memory Brain Activation Neuropsychological Tests	Female patients demonstrate different and more widespread working memory activation patterns, and these differences persist over time	73	Yes
Shao et al. 2018 [50] (China)	Observational: neuroimaging and neuropsychology	UC	32 patients with mTBI (≤24 h): 18 males and 14 females 30 healthy controls: 14 males and 18 females	Cortical Thickness: high-resolution MRI images Neurocognitive Assessments: Trail-Making Test Part A and Digit Symbol coding score, Forward Digit Span and Backward Digit Span, working memory, and executive function, Verbal Fluency Test for language ability, semantic memory, and executive function, PTSD Checklist-Civilian Version	Female patients showed a non-significant tendency of increased cortical thickness in the left insula cortex. Female patients had higher scores on the PTSD Checklist–Civilian	77	Yes Yes
Wang et al. 2018 [51] (China)	Cross-sectional: neuroimaging and neuropsychology	UC	54 Patients with mTBI (≤7 days post-injury): 27 males, 27 females 34 healthy control subjects: 17 males, 17 females	rs-fMRI Neuropsychological Assessments: Trail-Making Test Part A Digit Symbol Coding score, Forward Digit Span. Backward Digit Span, Verbal Fluency Test, semantic memory, and executive function PTSD Checklist–Civilian Version	A distinct pattern was observed in the right middle frontal gyrus and its connectivity with other brain regions in females, which may explain sex-specific clinical symptoms such as PTSD.	82	Yes
Wang et al. 2018 [52] (Taiwan)	Observational: neuropsychology	UC	192 Patients with mTBI (1 and 6 weeks post-injury): 61 male and 131 female	Self-reporting questionnaires: BAI BDI Blood samples	Male patients carrying T allele of rs6265 had significantly higher BAI scores in the first week (*p* = 0.01) and the sixth week (*p* = 0.038). They also showed higher BDI scores in the first week (*p* = 0.029) and the sixth week (*p* = 0.021)	68	Yes
					Female patients carrying the T allele had significantly higher BDI scores in the first week (*p* = 0.015), but this difference was not observed in the sixth week.		Yes
Bai et al. 2019 [53] (China)	Longitudinal: neuroimaging and neuropsychology	UC	41 Patients with mTBI (acute stage to 1 month post-injury): 25 male, 16 female 25 Healthy controls: 11 male, 14 female	Neuropsychological Tests: Rivermead Post-Concussion Symptom Questionnaire and PTSD Checklist civilian CBF (MRI)	Gender and the time following the injury (sub-acute phase) has a significant effect on the CBF in frontal cortex A reduced CBF observed in male mTBI patients correlated with poorer cognitive performance in subacute phase compared to the acute phase, a correlation not seen in female patients.	77	Yes Yes
Studenka et al. 2019 [54] (USA)	Cross-sectional: neuropsychology	UC	73 Concussion group (≥6 months post-injury): 51 males, 22 females 75 control group: 49 males, 26 females	seated visual-motor tracking task	Concussed females may be more susceptible to lasting alterations in visual-motor performance with increasing numbers of concussions	64	Yes
Yue et al. 2019 [55] (USA)	Prospective: neuroimaging and neuropsychology	UC	100 Patients with mTBI (6 months post-injury): 71 males and 29 females.	GOSE PCSS/PTSD RPQBrief Symptom Inventory Satisfaction with Life Scale Trail Making Test Wechsler Adult Intelligence Scale Fourth Edition, Processing Speed Index California Verbal Learning Test, Second Edition	Females had a significantly higher proportion of unfavourable GOSE outcomes, PCSS, lower quality of life, higher rates of clinically significant depression, and worse sleep quality at six months.	77	Yes
LeBlanc et al. 2021 [56] (Canada)	Retrospective: neuroimaging and neuropsychology	UC	128 Patients with mTBI (acute): 84 male and 44 female	CT scan Auditory comprehension: Boston Diagnostic Aphasia Examination. Verbal reasoning: Detroit Test of Learning Aptitude. Confrontation naming: Boston Naming Test Verbal fluency Conversational discourse: Protocole Montréal d’évaluation de la communication. Reading comprehension: (CAAT) and (TRF)	Females performed better on letter-category naming. Other cognitive-communication measures: males and females performed similarly in the acute stage	77	Yes
Levin et al. 2021 [16] (USA)	Prospective: neuropsychology	UC	2000 Patients with mTBI (2 weeks, 3 months, 6 months, and 12 months post-injury): 1331 male and 669 female Control: 299 patients with orthopaedic trauma: 199 male and 100	RPQ PTSD Checklist Patient Health Questionnaire Brief Symptom Inventory	Females exhibited higher levels of post-traumatic stress, depressive, anxiety, and somatization symptoms across all post-injury time points compared with males.	82	Yes
Pauhl et al. 2022 [57] (USA)	Observational: neuropsychology	UC	21 Patients with concussion (within 72 h, 1 week, and 2 weeks post-injury): 12 males and 9 females 21 controls: 12 males and 9 females	Motor evoked potential amplitude: to assess corticospinal excitability. Cortical silent period duration: to assess corticospinal inhibition. Electromyography: to record evoked potentials from the first dorsal interosseous muscle	Males had significantly longer Cortical Silent Period durations and this indicates greater corticospinal inhibition in males. No significant differences in motor-evoked potential amplitude or cortical silent period duration between males	91	Yes No
Anderson et al. 2023 [58] (Australia)	Retrospective: neuropsychology	UC	94 Patients with mTBI (1–4 days and 6–12 weeks post-injury): 71 males, 23 females	RPQ CCAMCHI Inventory of Depressive Symptomatology for depression, BAI for anxiety, PTSD Checklist for PTSD symptoms.	Female sex was associated with an increased likelihood of PCS reporting. Sex did not significantly predict CCAMCHI (subjective cognitive impairment)	82	Yes No
Anderson et al. 2023 [59] (Australia)	Prospective: neuropsychology	UC	92 Patients with mTBI (6–12 weeks post-injury): 69 males and 23 females	RPQ, Pittsburgh Sleep Quality Index, Multidimensional Fatigue Inventory, Psychological distress: (inventory of depressive symptomatology–self-report, BAI, and PTSD Checklist	Female sex is a significant predictor of PCS, even when accounting for the influence of fatigue and sleep disturbance and symptom reporting.	82	Yes
Wågberg et al. 2023 [60] (Sweden)	Prospective: neuropsychology	UC	595 Patients with mTBI (7–8 years post-injury): male 328 and female 267	RPQ GOSE	The symptom burden was higher in females and presented a worse GOSE outcome.	91	Yes
Levy et al. 2024 [2] (Australia)	Prospective: neuropsychology	UC	68 Patients with mTBI (6–10 weeks post-injury): 53 males and 15 females 40 Control group (trauma): 33 males and 7 females	RPQ CCAMCHI inventory of depressive symptomatology-self-report (BAI, and PTSD Checklist Short-Form McGill Pain Questionnaire Weschler Test of Adult Reading Symbol Digit Modalities Test Rey Auditory Verbal Learning Test	Female participants reported significantly higher levels of cognitive complaint, even after accounting for psychological distress and greater symptom burden.	77	Yes
Losoi et al. 2016 [61] (Finland)	Prospective: neuropsychology	UC	74 Patients with mTBI (1, 6 and 12 months post-injury): 45 male, 29 female 40 healthy controls: 20 male, 20 female	Symptoms: RPQ. GOSE PTSD-Checklist-Civilian Version Barrow Neurological Institute Fatigue Scale Insomnia Severity Index The Pain Subscale of the Ruff Neurobehavioral Inventory BDI- Second Edition The Resilience Scale QOLIBRI-OS The Rey Auditory Verbal Learning Test Return to work	Sex was not associated with chronic PCS.	91	No

Note: UC: Unclassified, ROB: Risk of Bias, PCSC: Post-Concussion Symptom Checklist, PCSS: Post-Concussion Symptoms Scale, mTBI: Mild Traumatic Brain Injury, RPQ: Rivermead Post Concussion Symptoms Questionnaire, GCS: Glasgow Coma Scale, MRI: Magnetic Resonance Imaging, PTSD: Posttraumatic Stress Disorder, rs-fMRI: Resting-state functional Magnetic Resonance Imaging, BAI: Beck Anxiety Inventory, BDI: Beck Depression Inventory, CBF: Cerebral Blood Flow, GOSE: Glasgow Outcome Scale Extended, QOLIBRI-OS: Quality of Life after Brain Injury–Overall Scale, CAAT: Canadian Adult Achievement Test, TRF: Test de Rendement pour Francophones, CCAMCHI: Cognitive Complaint After Mild Closed Head Injury.
